# Significance of cholecystectomy in cytoreductive surgery for advanced ovarian cancer

**DOI:** 10.1186/s12893-023-01956-1

**Published:** 2023-03-20

**Authors:** Joo-Hyuk Son, Su Ryeon Dong, Jisoo Kim, Jeeyeon Kim, Tae-Wook Kong, Suk-Joon Chang

**Affiliations:** Department of Obstetrics and Gynecology, Ajou University School of Medicine, 164 Worldcup-ro, Yeongtong-gu, 16499 Suwon, Republic of Korea

**Keywords:** Advanced ovarian cancer, Preventive cholecystectomy, Cytoreductive surgery

## Abstract

**Background:**

There have been no studies concerning the complications or benefits of cholecystectomy in ovarian cancer. In this study, we aimed to evaluate the outcomes of cholecystectomy performed during various time periods of the disease course and suggest a management strategy for cholecystectomy in ovarian cancer.

**Methods:**

We retrospectively reviewed the medical records of patients with advanced ovarian cancer who underwent cholecystectomy during the cytoreductive surgery from 2009 to 2020. Cholecystectomy was primarily indicated when the gallbladder and surrounding structures were considered to have metastatic tumor invasion. If the final pathologic results showed free of malignant tumor, patients were placed into the no-infiltration group. Clinical outcomes including the recurrence rate and complications were analyzed.

**Results:**

A total of 62 patients underwent cholecystectomy, 48 of whom (77.4%) underwent cholecystectomy during primary or interval debulking surgery, whereas 14 (22.6%) underwent cholecystectomy during the follow-up period (five with benign disease and 9 with disease recurrence). Among the patients, 32 (51.6%) patients were included in the no-infiltration group in the final pathology. There were no complications observed in the no-infiltration group (n = 32). Seven (78%) of the nine patients who received cholecystectomy for disease recurrence had metastatic disease in the porta-hepatis or lesser sac at the time of primary surgery. However, no recurrent lesions were observed around the porta-hepatis in patients who received cholecystectomy during primary treatment.

**Conclusion:**

Considering the safety of the procedure, as well as the risk of disease recurrence or cholecystitis, a cholecystectomy can be offered to patients with ovarian cancer who have metastatic lesions around the gallbladder and porta-hepatis at the time of primary surgery.

## Introduction

Ovarian cancer is the leading cause of gynecological cancer-related deaths worldwide [1]. Most patients with ovarian cancer are diagnosed at advanced stages because of inadequate screening tools and a lack of clinical symptoms. Cytoreductive surgery with platinum-based adjuvant chemotherapy has been the most effective therapeutic strategy in the treatment of advanced ovarian cancer [2–4]. Over the past few decades, the surgical approach has evolved toward radical surgeries because residual disease is the most important prognostic factor in ovarian cancer [2]. The main route in the spread of ovarian cancer is intraperitoneal seeding. Sometimes, the intraoperative judgment for tumor involvement of the gallbladder is very challenging. If it is after neoadjuvant chemotherapy (interval cytoreductive surgery), the lesions are more difficult to judge if it has definite tumor invasions [5, 6]. Despite upper abdominal surgeries being widely performed in patients with ovarian cancer, there are few studies describing the approach for gallbladder lesions and cholecystectomy in these patients.

Cholecystectomy performed at the time of primary surgery may be beneficial considering that subsequent cholecystectomy is more challenging after upper abdominal surgery and has an increased risk for bile duct injuries and a longer operation time [7, 8]. Further, concomitant cholecystectomy might reduce the risk of disease recurrence around the gallbladder. However, the potential benefits of prophylactic cholecystectomy performed concomitantly with non-biliary abdominal surgery, especially without definite cancer invasion, are debatable.

Prophylactic cholecystectomy has been evaluated in several different diseases, including gastrointestinal tract diseases and hematologic diseases [9–14]. Most studies report these prophylactic procedures as feasible and safe. In some studies, the overall mortality of cholecystectomy is reported as minimal or even zero [10, 15]. However, morbidity is variable, ranging from simple complications to bile duct injuries with varying degrees of complexity [16]. Moreover, there have been no studies concerning the complications or benefits of cholecystectomy in ovarian cancer.

In this study, we aimed to evaluate the clinical outcomes of cholecystectomy performed during various time periods of the disease course and suggest a management strategy for cholecystectomy in advanced ovarian cancer.

## Materials and methods

This retrospective study included patients with ovarian cancer who underwent cholecystectomy as a part of cytoreductive surgery from May 2009 to April 2020 in Ajou University Hospital, Suwon, South Korea. A total of 62 patients were identified as having undergone cholecystectomy during their disease course. Patients were included consecutively regardless of the timing of the cholecystectomy (primary debulking surgery, interval debulking surgery, or at the time of disease recurrence). Informed consent was waived due to the retrospective nature of this study. The study protocol was approved by the institutional review board of Ajou University Hospital (AJIRB-MED-MDB-21-116).

Cholecystectomy was primarily indicated when the gallbladder and its surrounding structures were considered to have metastatic tumor invasion during the primary, interval, or secondary cytoreductive surgery. In cases where benign disease of the gallbladder was noted in preoperative computed tomography (CT), cholecystectomy was performed concomitantly. In cases of benign disease during the follow-up times, the cholecystectomy was performed laparoscopically. All the procedures were performed by biliary surgeons or an experienced gynecologic oncologic surgeon during the study period. If final pathologic results showed free of malignant tumor, patients were placed into the no-infiltration group.

Patients were classified into two groups according to the timing of the cholecystectomy: at the time of primary disease or during follow-up (benign cause or recurrent disease). Complications related to cholecystectomy were analyzed in all patients. A subgroup analysis of patients who experienced disease recurrence was performed, and its relation to the initial lesion was reviewed. Also, the disease recurrence patterns of the patients who underwent cholecystectomy during the primary treatment were analyzed.

Clinical characteristics included age, FIGO stage, timing of the surgery, pathologic results, complications, and residual disease status. The reason for the surgery and the initial porta-hepatis surgery were analyzed in patients with disease recurrence (the follow-up group). Lastly, recurrence patterns of patients who underwent cholecystectomy during the initial surgery were analyzed. Descriptive statistics were used to characterize the patient population and the type of intestinal surgery. Statistical analysis was performed using IBM SPSS Statistics for Windows (version 25.0, IBM Corp., Armonk, NY, USA).

## Results

A total of 62 patients with ovarian cancer underwent cholecystectomy during the study period. The patients’ median age was 52 years old (range, 24–78 years). Most patients had FIGO stage IIIC-IV disease. Forty eight (77.4%) patients underwent cholecystectomy at the time of primary debulking surgery (PDS = 32) or the interval debulking surgery (IDS = 16), and 14 (22.6%) patients underwent cholecystectomy during the follow-up period. Although suggestive of metastatic lesions, pathologic lesions were not confirmed in 32 (51.6%) patients in the final pathology and they were categorized into the no-infiltration group. Of all patients, procedural complications occurred in one (1.6%) patient with gallbladder bed bleeding. There were no cases with re-admission in 30 days after the surgery. Time to the initiation of chemotherapy was median 22 (18–51) days. There was no surgical morbidity throughout the follow-up period in the no-infiltration group (n = 32; Table 1).

**Table 1:** Characteristics of patients with ovarian cancer who underwent cholecystectomy during the course of the disease (n = 62)

Age (years)	52 (24–78)
Initial FIGO stage	
I	3 (4.8%)
II	0 (0%)
III	43 (69.4%)
IV	16 (25.8%)
Time of the cholecystectomy	
Primary debulking surgery	32 (51.6%)
Interval debulking surgery	16 (25.8%)
Secondary debulking surgery	9 (14.5%)
Follow-up (only cholecystectomy)	5 (8.1%)
Final pathology	
No lesion (no infiltration group)	32 (51.6%)
Cancer invasion	18 (29.0%)
Chronic cholecystitis	12 (19.4%)
Complication after cholecystectomy	
GB bed bleeding	1 (1.6%)
Drain placement in 30 days	1 (1.6%)
Readmission in 30 days	0 (0.0%)
Time to chemotherapy (days)	22 (18–51)
Residual disease after primary surgery	
NGR	45 (72.6%)
GR-1	16 (25.8%)
GR-B	1 (1.6%)
Follow-up duration, month	18 (2–107)

Data are presented as median (range) or n (%)

*FIGO* The International Federation of Gynecology and Obstetrics, *GB* gall bladder, *NGR* no gross residual disease, *GR-1* gross residual disease size < 1 cm in maximal diameter, *GR-B* gross residual-bulky for residual disease size > 1 cm in maximal diameter

There were 14 patients who underwent cholecystectomy during follow-up, five of whom (35.7%) underwent cholecystectomy due to benign disease (cholecystitis) of the gallbladder and nine (64.3%) due to disease recurrence. Although all procedures were attempted laparoscopically in patients with cholecystitis (n = 5), one patient underwent open surgery because of severe adhesions. Among the patients with recurrent disease (n = 9), most patients (7/9, 78%) had metastatic disease in the porta-hepatis or ligamentous attachments around the gallbladder at the time of primary surgery (Table 2).

**Table 2:** Analysis of cholecystectomy during follow-up (n = 14)

Age, years	53 (39–66)
Initial FIGO stage	
I	2 (14.3%)
III	10 (71.4%)
IV	2 (14.3%)
Reason for the surgery	
Disease recurrence	9 (64.3%)
Benign disease of GB	5 (35.7%)
Existence of metastatic lesion in the porta- hepatis at the time of primary surgery in cases with disease recurrence (n = 9)	
Yes	7 (77.8%)
No	2 (22.2%)

Data are presented as median (range) or n (%)

*FIGO* The International Federation of Gynecology and Obstetrics, *GB* gall bladder

Recurrence patterns were analyzed in patients who underwent cholecystectomy during primary treatment (PDS or IDS, n = 48). Twelve patients experienced disease recurrence during follow-up. There were no recurrent lesions observed around the gallbladder bed and porta-hepatis in patients who received cholecystectomy during primary treatment (Table 3).

**Table 3:** Recurrence patterns of patients who underwent cholecystectomy during the primary treatment (PDS or IDS, n = 48)

Age	53 (24–78)
FIGO stage	
I	1 (2.0%)
II	0 (0.0%)
III	33 (68.8%)
IV	14 (29.2%)
Residual disease status after primary treatment	
NGR	33 (68.8%)
GR-1	14 (29.2%)
GR-B	1 (2.1%)
Number of recurrences	12 (25%)
Location of recurrence	
Lymph node	5 (10.4%)
Peritoneum	3 (6.3%)
Bowel mesentery	2 (4.2%)
Liver	2 (4.2%)
Lung	1 (2.4%)
Bone	1 (2.4%)
Porta-hepatis	0 (0%)

Data are presented as median (range) or n (%)

*FIGO* The International Federation of Gynecology and Obstetrics, *NGR* no gross residual disease, *GR-1* gross residual disease size < 1 cm in maximal diameter, *GR-B* gross residual-bulky for residual disease size > 1 cm in maximal diameter

## Discussion

To the best of our knowledge, this is the first study to report on significance of cholecystectomy in patients with ovarian cancer. The hepatobiliary involvement in ovarian cancer is often associated with high tumor load and could require high complex multi-visceral surgery [17]. Also, the tumor lesions in gall bladder fossa or porta hepatis is considered as the second most common sites as the missed recurrence sites [18]. Since these lesions are one of the main obstacles in complete resection in primary cytoreductive surgery, neoadjuvant chemotherapy is frequently adopted. However, neoadjuvant chemotherapy has been reported to cause observable microscopic or macroscopic changes such as tumor necrosis, fibrosis, and tumor-induced inflammation. Notably, these lesions can interfere with the evaluation of tumor extent more difficult [5, 6]. In this study, cholecystectomy was mainly performed if a gallbladder and its surrounding structures were considered to have metastatic tumor invasion. The complication rate related to the procedures was relatively low; there were no complications, especially in the no- infiltration group. Most patients who experienced cancer recurrence in the gallbladder had metastatic lesions around the porta-hepatis or ligamentous attachments around the gallbladder at the time of primary surgery. Considering patients are exposed to a concomitant risk for developing cholecystitis or disease recurrence with increased subsequent surgical difficulty, a cholecystectomy might be offered to patients who have metastatic disease around the gallbladder at the time of primary surgery.

Although upper abdominal surgeries are widely performed in patients with ovarian cancer, there are few studies concerning cholecystectomy in patients with ovarian cancer. Thomsen et al. reported the risk for cholecystitis in patients with cancer [19]. They evaluated 51,228 patients with incident cancer compared with 512,280 age‐matched people in the general population. In that study, patients with cancer generally had a higher risk of developing cholecystitis than the general population throughout the follow‐up period. The relative risk (RR) for cholecystitis among patients with ovarian cancer compared with the general population cohort was 1.38 (95% confidence interval [CI], 0.19–9.87). The RR for cholecystitis doubled during the first 6 months after cancer diagnosis (RR = 1.95; 95% CI, 1.50–2.54), after which the RR declined but remained > 1 throughout the follow‐up period (RR = 1.23; 95% CI, 1.05–1.45). Moreover, patients between the ages of 51 and 70 years had the highest risk for cholecystitis compared with other age groups. Although evidence for this association is lacking, cancer might increase the risk for cholecystitis due to several mechanisms, including malnutrition and rapid weight loss, chemotherapy‐induced neutropenic episodes, and radical surgeries (including bowel resection) [20–22]. As all of these factors may occur in patients with ovarian cancer, there may be significant clinical implications for the increased risk of cholecystitis in patients with ovarian cancer.

Cholecystectomy is considered a safe procedure, and the overall mortality is reported as minimal or even zero in many studies [10, 15]. Prophylactic cholecystectomy has been evaluated among other disease entities, including gastrointestinal tract diseases (gastric cancers, colorectal surgery, short bowel syndrome, and gastric bypass surgery) and hematologic diseases (sickle cell anemia) [9–14, 19, 23]. Most studies report prophylactic procedures as feasible and safe. Marco et al. performed a randomized controlled trial comparing the preoperative complications or costs of prophylactic cholecystectomy [10]. A total of 130 patients were randomized (prophylactic cholecystectomy, n = 65 vs. standard surgery, n = 65); patients who underwent prophylactic cholecystectomy did not experience any additional perioperative complications related to the biliary surgery. There were no additional mortality risks in the prophylactic cholecystectomy group. After a median follow-up of 62 months, long-term follow-up results of patients confirmed the safety of prophylactic cholecystectomy, although there was no significant impact on the natural course of the patients [9]. In our study, although one patient experienced perioperative complications, there were no complications related to the procedure in the no-infiltration group.

The potential benefit of prophylactic cholecystectomy performed concomitantly with non-biliary abdominal surgery, especially without definite cancer invasion, are debatable. However, abdominal surgeries are reported to be a risk factor for the development of gallstones or related complications [23]. Numerous factors might increase the risk for cholecystitis, especially in ovarian cancers, including prolonged parenteral nutrition with decreased diet; blood transfusions during postoperative or chemotherapeutic periods; opiate medications, which decrease gall bladder motility and increase the risk of bile stasis; and bowel resection, which might affect alteration of the enter-hepatic circulation of bilirubin. Additionally, our data showed a high risk of cancer invasion in patients with lesions around the gallbladder at the time of primary surgery. Considering the low complication rates of preventive group and subsequent surgical exploratory difficulty, cholecystectomy can be offered in patients with advanced ovarian cancer presenting with metastatic lesions around the gallbladder.

Some limitations of the present study should be acknowledged. First, the retrospective nature of the study might inherently induce patient or treatment selection bias. Second, our study had a small sample size with no comparative patients for the preventive group, and therefore our findings should be confirmed in a larger patient cohort. Third, long-term follow-up data and risk factors for the recurrence or subsequent cholecystitis could not be confirmed. However, our study recruited consecutive patients in a single institution with the same treatment indications during the study period. Furthermore, these data can be a practical reference for upper abdominal cytoreductive surgery in ovarian cancer when cholecystectomy is considered.

## Conclusion

Cholecystectomy has a minimal surgical complication risk in patients with ovarian cancer. Considering the risk of recurrence or benign disease of the gallbladder, a cholecystectomy might be indicated in patients with cancerous lesions around the gallbladder at the time of primary surgery.

## Data Availability

The datasets used during the current study are available from the corresponding author on reasonable request.

## Abbreviations

FIGO: International Federation of Gynecology and Obstetrics
PDS: Primary debulking surgery
IDS: Interval debulking surgery
